# Central Aortic Pressure and Arterial Stiffness in Parkinson's Disease: A Comparative Study

**DOI:** 10.1155/2022/6723950

**Published:** 2022-07-12

**Authors:** Mehmet Balal, Meltem Demirkiran, Saime Paydas

**Affiliations:** ^1^Çukurova Univeristy, Medicine School, Department of Neurology, Adana 01031, Turkey; ^2^Çukurova Univeristy, Medicine School, Department of Nephrology, Adana 01031, Turkey

## Abstract

**Background:**

Cardiovascular autonomic dysfunction, which leads to hemodynamic disorders, is commonly observed in patients with Parkinson's disease (PD). Central aortic pressure (CAP) is the systolic blood pressure (SBP) at the root of the aorta. In young people, CAP is lower than peripheral arterial blood pressure. In older people, the difference between CAP and peripheral arterial blood pressure decreases depending on the extent of arterial stiffness (AS). In patients with AS, CAP increases. CAP is thus regarded as an indicator of AS.

**Objective:**

To compare CAP and other hemodynamic parameters for AS between patients with Parkinson's disease and control group. We also aimed to evaluate changes in these hemodynamic parameters after the levodopa (LD) intake.

**Methods:**

We included 82 patients with PD and 76 healthy controls. Age, sex, disease duration, disease subtype, Hoehn–Yahr stage (H&Y), and nonmotor symptoms (NMS) were documented. TensioMed Software v.3.0.0.1 was used to measure CAP, peripheral arterial blood pressure, pulse pressure (PP), heart rate (HR), mean arterial pressure (MAP), augmentation index (AI), pulse wave velocity, and ejection time. All patients were being treated with LD, and measurements were performed 1 h before and 1 h after LD intake.

**Results:**

Baseline peripheral arterial blood pressure and CAP values were significantly higher in the PD group than in the control group (*p* < 0.001 and *p*=0.02, respectively). Most cardiac hemodynamic parameters, including peripheral arterial blood pressure and CAP, decreased significantly (*p* < 0.02 and *p* < 0.001, respectively) after LD intake in the PD group. Disease subtype, duration, and severity did not affect any of the hemodynamic parameters. When NMS were evaluated, patients with psychosis and dementia showed higher baseline parameters.

**Conclusion:**

Loss of postganglionic noradrenergic innervation is well-known with PD. Several cardiac hemodynamic parameters were affected, suggesting cardiac autonomic dysfunction in these patients. The data obtained were independent of disease severity, duration, and subtype. After LD intake, most of these parameters decreased, which might have a positive effect on the vascular burden.

## 1. Introduction

Over the past two decades, strong evidence has identified loss of cardiac noradrenergic neurons in patients with Parkinson's disease (PD). Cardiac sympathetic neuroimaging has demonstrated a loss of postganglionic noradrenergic innervation in patients with PD. Accumulating data have drawn attention to the cardiac autonomic involvement of the disease, and studies have multiplied in the succeeding years [1, 2].

Nonmotor symptoms (NMS) are common during the progression of PD. Cardiovascular autonomic dysfunction such as orthostatic hypotension (OH), labile tension, nocturnal hypertension, and reduced heart rate (HR) variability can be observed in patients with PD. Cardiovascular autonomic dysfunction in patients with PD can lead to microvascular disorders that contribute to cerebral perfusion defects [3]. Vascular damage can lead to hemodynamic disorders and dementia [3–5]. Arterial stiffness (AS) is an important indicator of vascular damage. There are limited data on AS in patients with PD, and central aortic pressure (CAP) is an indicator of AS; it is measured via special gadgets on the brachial artery [5–8].

CAP is the systolic blood pressure (SBP) at the root of the aorta. In young people, CAP is lower than peripheral blood pressure owing to the elasticity of the aorta. In older adults, the difference between CAP and peripheral arterial blood pressure decreases, and values become closer to each other [8]. Several studies have demonstrated that it is better for heart health to have a lower CAP than peripheral arterial blood pressure [8–10]. A few other parameters can be used to assess AS [11, 12]. These are the augmentation index (AI) and pulse pressure (PP). AI is defined as the proportion of central PP owing to the late systolic peak, which is attributed to the reflected pulse wave (PW) [11–15]. AI shows peripheral vascular resistance and possible endothelial damage, and its increase is considered an early sign of atherosclerosis. PP is the difference between systolic and diastolic pressures; values > 60 mm·Hg are considered signs of cardiovascular risk [16].

Cardiovascular autonomic changes in patients with PD and the effect of levodopa (LD) have been evaluated in several studies [3, 5–8]. The vasodepressor effect is well-known, and its combination with a dopa-decarboxylase enzyme inhibitor reduces the peripheral effects of LD [6]. To our knowledge, there are no data in the literature regarding the effects of LD on CAP.

Previous studies have shown that central hemodynamic disorders lead to AS in patients with PD [17, 18]. We hypothesized that CAP is affected by PD and may be used as an indicator of AS in these patients. We aimed to investigate CAP and the other hemodynamic parameters for AS described above in the control group and patients with PD, in relation to different subtypes of PD and their relation to other NMS. As a secondary endpoint, we aimed to evaluate these parameters before and after the administration of LD.

## 2. Material and Methods

### 2.1. Participants

This comparative study was conducted at the Movement Disorders Unit of the Çukurova University Neurology Department. Informed consent was obtained from all participants, and the study was approved by the local ethics committee. One hundred and fifty-eight participants (82 patients and 76 controls) were included in the study. The 82 patients had been diagnosed with idiopathic PD according to the United Kingdom Brain Bank criteria. Patients with Parkinson's plus syndromes, secondary parkinsonism, and PD who were not on LD treatment were excluded. The control group consisted of 76-age- and sex-matched healthy controls. Those with hypertension, heart disease, diabetes mellitus, and those who smoked or used any antihypertensive drugs were not included in either group. Age, sex, and body mass index (BMI) were recorded for both groups. Patients were grouped according to disease duration into those with five or fewer years and those with more than five years. For disease severity, patients were divided into two groups: those with a Hoehn and Yahr score (H&Y) < 3 and those with a H&Y score ≥ 3. The presence of other NMS (OH, incontinence, dementia, and psychosis) was assessed in each patient. Patients were also separated into akinetic rigid (ARP) and tremor dominant subtypes (TDP).

### 2.2. Peripheral and Central Aortic Pressure Measurements

An arteriograph (TensioMed software v.3.0.0.1, Sun Solutions, Hungary) was used to record hemodynamic data, including CAP, SBP, DBP, PP, AI, HR, and mean arterial pressure (MAP). The device used in this study automatically measures and records hemodynamic parameters. Participants were informed of the study and the procedures involved. The hemodynamic parameters of all participants were measured on their right arm. The size of the cuff was 15 × 55 cm. All measurements in both groups were made at eight o'clock in the morning. Measurements were performed in a quiet room. Postrest measurements were performed in both groups in the supine position after a minimum 10-minute rest. In the patient group, measurements were carried out an hour before and an hour after the first dose of 100 mg levodopa/25 mg benserazide in the morning before the administration of any other drug.

### 2.3. Statistical Analysis

All analyses were performed using IBM SPSS Statistics version 20.0 statistical software package. Categorical variables were expressed as numbers and percentages, whereas continuous variables were summarized as mean and standard deviation, or as median and minimum-maximum where appropriate. The chi-squared test was used to compare categorical variables between the groups. The normality of distribution for continuous variables was confirmed using the Shapiro–Wilk test. For comparison of continuous variables between the two groups, the Student's *t*-test or Mann–Whitney *U* test was used depending on whether or not the statistical hypotheses were fulfilled. For comparison of two related (paired) continuous variables, the paired sample *t*-test or Wilcoxon-signed rank test was used depending on whether or not the statistical hypotheses were fulfilled. Correlation analysis for the presence of NMS was performed between the PD subtypes. The statistical significance level for all tests was set at *p* < 0.05.

## 3. Results

There were no significant differences between patients with PD and controls in terms of age, sex, and BMI. The mean duration of disease was 7.04 ± 4.22 years. The mean H&Y score was 2.56 ± 0.86. The demographic data are shown in Table 1.

**Figure 1:**
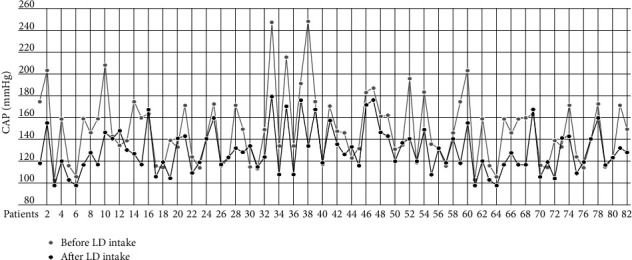
Central aortic pressure before and after the levodopa intake in Parkinson's disease. CAP :  central aortic pressure; LD :  levodopa.

Age, sex, disease duration, and H&Y stage were not correlated with any of the hemodynamic parameters. SBP, PP, HR, and CAP values were significantly higher in PD patients than those in the control group (*p*1) before LD intake. Diastolic blood pressure (DBP) and AI were also higher, but the differences were not statistically significant. However, after administration of LD, hemodynamic values changed and SBP, DBP, PP, AI, and CAP reduced significantly without any effect on HR (*p*1) (Table 2 and Figure 1). Interestingly, in the PD group, most of the values (SBP, DBP, AI, and CAP) reached levels even lower than those in the control group after LD administration.

**Figure 2:**
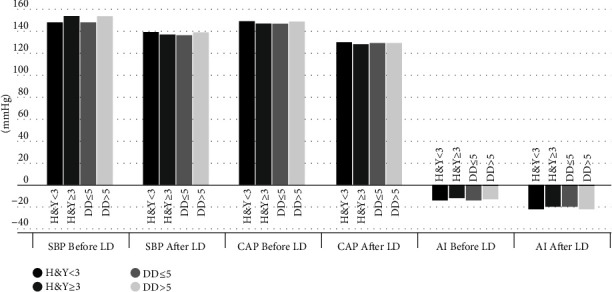
Hemodynamic effects of levodopa on systolic blood pressure, central aortic pressure and augmentation index in relation to disease severity and duration. AI : augmentation index, CAP : central aortic pressure, DD : disease duration, H&Y : Hoehn and Yahr score, LD : levodopa, SBP : systolic blood pressure.

When the changes in hemodynamic variables after LD intake were examined in relation to disease severity, the results obtained in patients with mild-to-moderate (H&Y < 3; *p*1) and those with more severe disease (H&*Y* ≥ 3; *p*2) were similar. There was a significant reduction in SBP, DBP, PP, AI, and CAP in both groups (Table 3 and Figure 2). HR increased slightly after LD intake in patients with the H&Y stage < 3. HR did not change after LD intake in patients with the H&Y stage ≥ 3. Similar hemodynamic changes were observed after LD intake; significant reductions in SBP, DBP, PP, AI, and CAP without any effect on HR were observed in patients with disease durations ≤5 years and >5 years (*p*_*3*_ and *p*_*4*_) (Table 3 and Figure 2). The differences in baseline hemodynamic parameters between patients with less severe and more severe disease, as well as between patients with short and longer disease duration, were not statistically significant.

Subgroup analysis was performed among patients with different PD subtypes and with and without NMS (Table 4). When PD subtypes were considered, SBP and PP were significantly higher in patients with ARP. AI was lower in the ARP group than in the TDP group. CAP was not affected by the disease subtype, and the values were similar (Table 4). After LD intake, SBP, DBP, AI, and CAP decreased significantly in the TDP group (Table 4). In the ARP group, after LD intake, the same parameters decreased, except for AI, which was already low in this subtype.

When the presence of other NMS was considered, most of the hemodynamic parameters, including AI and CAP, were higher in patients with incontinence and constipation than in patients without these NMS. In patients with OH, the only significant difference was in AI, which was higher than that in patients without OH (Table 4). In all groups with or without autonomic dysfunction, there was a significant reduction in most hemodynamic parameters, including CAP, SBP, DBP, PP, and AI after LD intake (Table 4).

Patients with psychosis had lower baseline hemodynamic parameters than those without psychosis (Table 4). In patients without psychosis, there was a significant reduction in all hemodynamic parameters after LD intake, whereas in patients with psychosis, there was no change in any of the parameters, except for a significant reduction in CAP after LD administration (Table 4).

All baseline parameters were higher in patients with dementia than in those without dementia; however, CAP and AI did not reach statistical significance (Table 4). In patients with dementia, SBP, DBP, and CAP decreased after LD administration, whereas in patients without dementia, all hemodynamic parameters decreased after LD intake (Table 4).

## 4. Discussion

In our study, both CAP and peripheral arterial blood pressure values, as well as other cardiac hemodynamic variables (PP and AI), were higher in the PD group than in the control group. Age, sex, disease duration, and disease severity did not affect any of these parameters. There was a significant decrease in most hemodynamic parameters after LD intake, except for HR. In subgroup analysis, SBP and PP were significantly higher in the ARP group than in the TDP group. In patients with or without autonomic dysfunction, there was a significant reduction in most hemodynamic parameters, including CAP, SBP, DBP, PP, and AI after LD intake.

High systolic and DBP may lead to increased microvascular damage, atherosclerosis, cardiac disease, and cerebral perfusion defects [16]. Several authors have suggested that an increase in vascular burden may lead to increased dopaminergic loss [19, 20]. However, the results of our study do not support this suggestion because cardiac hemodynamic data did not change with age, sex, severity, or duration of the disease. Our results suggest that cardiac autonomic deficiency may be independent of motor symptom progression in patients with PD.

There was a significant decrease in most hemodynamic parameters after LD intake, except for HR. The more pronounced decline in CAP, AI, and peripheral arterial blood pressure indicated a decrease in peripheral arterial resistance. In our study, CAP, PABP, and AI reached even lower values than those of the control group after LD intake. Moreover, its effect was independent of disease severity, duration, and subtype. Several studies have shown that a CAP lower than peripheral arterial blood pressure is healthier for the heart [3–5]. Furthermore, an increase in AI is considered an early sign of atherosclerosis. Therefore, LD may enable a reduction in the atherosclerotic process by causing a decrease in these parameters. However, the limited number of participants and uncontrolled effects of other dopaminergic and nondopaminergic drugs complicate the evaluation of the effect of LD on these parameters. Therefore, answering this question is beyond the scope of this study. Longitudinal studies with controlled variables may provide answers to this question.

In subgroup analysis, SBP and PP were significantly higher in the ARP group than in the TDP group. The ARP subtype may be more prone to cardiovascular disease than the TDP subtype, and this finding is in accordance with several other studies [21–23]. However, this issue is controversial, and there are a few reports on TDP subtypes with more severe impairment of the cardiac sympathetic system than the ARP subtypes; this was later negated in a slightly larger study by the same authors [24, 25]. Furthermore, the AI was significantly lower in the ARP group in our study. A study conducted by Chiaravalloti et al. observed that in sympathetic myocardial scintigraphy, myocardial uptake was significantly more impaired in ARP subtypes than in TDP subtypes at the same stage of the disease. Another interesting finding was the very low AI value in patients without autonomic NMS (incontinence, constipation, OH, and dementia) and low CAP in patients without incontinence and constipation. Patients with lower autonomic involvement had higher AI and CAP values. Perhaps, another PD subtype should be considered with more prominent autonomic involvement, independent of disease duration and severity, as well as the known subtypes. This subgroup might resolve discrepancies in the literature regarding this issue. The presence of other autonomic dysfunctions, incontinence, constipation, and OH was correlated with higher AI values. Both SBP and CAP were higher in patients with incontinence and constipation.

In patients with psychosis, the hemodynamic parameters of cardiac dysfunction were lower than those in patients without psychosis. This may be due to the use of antipsychotic medications to treat these patients. All hemodynamic variables were higher in patients with dementia than in those without but not all reached statistical significance. In fact, this may not be a reason but a result. According to Pierzchlinska et al, variability in hemodynamic parameters initiates the occurrence of dementia, causing white matter hyperintensity and microvascular damage [26]. The results in these subgroups with psychosis and dementia should be interpreted with caution because of the small number of patients included.

The sympathetic noradrenergic denervation observed in patients with PD seems to occur independently of striatal dopaminergic denervation [15, 26–28]. Our data confirm that neither the duration nor the severity of disease had any effect on cardiac hemodynamic parameters, suggesting that cardiac autonomic disorders are caused by mechanisms other than central causes. Jain and Goldstein [29] proposed three mechanisms for cardiovascular autonomic dysfunction in PD. The first is the loss of cardiac noradrenergic sympathetic nerves, which occurs in almost all PD patients regardless of the severity and duration of the disease. The second mechanism is extracardiac noradrenergic denervation, which is less severe than the cardiac noradrenergic challenge associated with PD [30]. The third is arterial baroreflex failure. Cardiac autonomic insufficiency is even suggested as a potential biomarker in the premotor period of PD [16, 17, 19]. If this is the case, providing procedures or parameters for early detection of cardiac autonomic dysfunction in patients with PD will be crucial in the upcoming years. A high PP (>63 mm·Hg) has already been suggested as a marker of preclinical cardiovascular disease rather than a sign of cardiovascular risk [31].

Our study had several limitations. We included a small number of participants, especially those with PD subtypes. Patients who were LD-naive were excluded from the study. The effects of other medications could not be excluded. Post hoc sample size calculations were not performed.

## 5. Conclusion

Loss of postganglionic noradrenergic innervation is well-known in patients with PD. In our study, several cardiac hemodynamic parameters, such as CAP and AS, were affected, suggesting more severe cardiac autonomic dysfunction in these patients. After LD intake, there was a decrease in most of these parameters, which may have a positive effect on the vascular burden. Further research is required to clarify this suggestion. Further studies are needed to answer the questions and suggestions raised in this study.

## Figures and Tables

**Table 1:** Demographic data of the controls and patients in relation to disease subtypes and NMS.

	Female	Male	Age	H&Y	DD	BMI
Control group(n: 76)	30 (39.5%)	46 (60.5%)	59.67 ± 11.02 (34–79)	—	—	27.02 ± 3.53 (19.10–35.40)
Parkinson's disease (n: 82)	37 (45.1%)	45 (54.9%)	60.56 ± 13.79 (29–83)	2.56 ± 0.86 (1–5)	7.04 ± 4.22 (2–30)	28.96 ± 4.16 (18.30–37.50)
H&Y < 3 (n: 42)	19 (45.2%)	23 (54.8%)	56.75 ± 14.57 (36–83)	—	4.34 ± 2.08 (2–9)	27.22 ± 4.04 (21.90–37.50)
H&Y ≥ 3 (n: 40)	18 (45.0%)	22 (55.0%)	64.19 ± 12.09 (29–81)	—	9.89 ± 4.46 (6–30)	29.80 ± 3.56 (18.30–36.10)
DD ≤ 5 year (n: 37)	18 (48.6%)	19 (51.4%)	58.66 ± 14.45 (35–83)	1.76 ± 0.56 (1–4)	—	26.27 ± 4.11 (18.30–37.50)
DD > 5 year (n: 45)	19 (42.2%)	26 (57.8%)	62.86 ± 12.76 (29–81)	2.89 ± 1.04 (2–5)	—	31.18 ± 3.64 (19.10–35.90)
Tremor dominant PD (n: 38)	20 (52.6%)	18 (47.4%)	59.94 ± 15.00 (29–83)	2.52 ± 0.79 (1–4)	8.00 ± 4.87 (3–30)	25.28 ± 3.90 (18.30–37.50)
Akinetic-rigid PD (n: 44)	26 (59.0%)	18 (41.0%)	61.09 ± 12.82 (34–78)	2.56 ± 0.91 (1–5)	6.22 ± 3.40 (2–13)	26.18 ± 3.64 (20.10–36.90)
Incontinence (+) (n: 21)	13 (61.9%)	8 (38.1%)	69.52 ± 13.16 (56–83)	2.65 ± 0.76 (2–5)	7.14 ± 3.57 (6–30)	28.41 ± 3.86 (18.30–35.78)
Incontinence (−) (n: 61)	24 (39.3%)	37 (60.7%)	57.47 ± 12.70 (29–76)	2.34 ± 0.86 (1–4)	6.36 ± 4.63 (2–16)	29.45 ± 4.56 (21.34–37.50)
Constipation (+) (n: 45)	19 (42.2%)	26 (57.8%)	64.43 ± 12.68 (44–83)	2.63 ± 0.84 (2–5)	7.25 ± 4.35 (3–30)	27.28 ± 4.67 (21.40–37.50)
Constipation (−) (n: 37)	18 (48.6%)	19 (51.4%)	54.19 ± 13.36 (29–78)	2.41 ± 0.86 (1–4)	6.70 ± 4.03 (2–14)	25.18 ± 4.41 (18.30–36.90)
Orthostatic hypotension (+) (n:31)	14 (45.1%)	17 (54.2%)	64.70 ± 13.26 (48–83)	2.69 ± 0.87 (1–5)	8.03 ± 5.05 (4–30)	26.41 ± 5.86 (18.30–35.78)
Orthostatic hypotension (−) (n:51)	23 (45.0%)	28 (55.0%)	58.03 ± 13.62 (29–77)	2.47 ± 0.84 (1–4)	6.45 ± 3.54 (2–16)	28.55 ± 4.98 (19.30–37.50)
Psychosis (+) (n: 12)	5 (41.6%)	7 (58.4%)	62.61 ± 12.11 (54–83)	2.88 ± 1.02 (2–5)	10.88 ± 5.55 (4–30)	27.62 ± 4.89 (20.40–37.50)
Psychosis (−) (n: 70)	32 (45.7%)	38 (54.3%)	53.88 ± 15.95 (29–77)	2.31 ± 0.83 (1–4)	6.57 ± 4.07 (2–18)	26.18 ± 4.41 (18.30–35.90)
Dementia (+) (n: 11)	5 (45.4%)	6 (54.6%)	72.27 ± 8.43 (59–83)	2.75 ± 0.58 (2–5)	6.54 ± 4.69 (6–30)	28.365 ± 4.89 (18.30–33.78)
Dementia (−) (n: 71)	32 (45.1%)	39 (54.9%)	58.74 ± 13.61 (29–74)	2.30 ± 0.89 (1–4)	7.12 ± 4.49 (2–16)	30.45 ± 4.32 (22.34–37.50)

(+): present; (−): absent.

**Table 2:** Hemodynamic data of participants.

	Control group	PD (before LD)	PD (after LD)	*p*1*-*value	*p*2*-*value
SBP (mm·Hg)	141.15 ± 20.25	151.64 ± 31.39	138.21 ± 18.94	**0.01**	≤**0.001**
DBP (mm·Hg)	82.97 ± 12.03	87.36 ± 18.72	77.58 ± 13.47	NS	≤**0.001**
PP (mm·Hg)	58.12 ± 12.86	64.37 ± 17.16	60.75 ± 12.82	**0.01**	**0.03**
HR (minute)	73.01 ± 9.40	77.10 ± 12.70	78.80 ± 12.80	**0.02**	NS
AI (%)	−16.49 ± 26.68	−13.83 ± 28.74	−21.60 ± 25.61	NS	**0.01**
CAP (mm·Hg)	137.98 ± 23.83	148.49 ± 31.52	129.60 ± 20.70	**0.02**	≤**0.001**

AI: augmentation index; CAP: central aortic pressure; DBP: diastolic blood pressure; HR : heart rate; LD: levodopa; NS: nonsignificant; PP: pulse pressure; SBP : systolic blood pressure. *p*_*1*_; control group vs PD before LD intake; *p*_*2*_: PD before LD vs PD after LD intake. Bold numbers indicate significance.

**Table 3:** Hemodynamic data according to disease severity and duration in PD patients.

	H&Y < 3		H&Y ≥ 3		*p*1*-*value	*p*2*-*value
	Before LD	After LD	Before LD	After LD		
SBP (mm·Hg)	148.54 ± 24.55	139.07 ± 18.95	154.90 ± 37.31	137.32 ± 19.14	≤**0.001**	≤**0.001**
DBP (mm·Hg)	83.92 ± 13.70	77.47 ± 13.93	90.97 ± 22.47	77.70 ± 13.14	**0.01**	**0.01**
PP (mm·Hg)	64.61 ± 14.65	61.83 ± 13.29	64.12 ± 19.64	59.62 ± 12.37	≤**0.001**	**0.02**
HR (minute)	75.35 ± 12.36	78.69 ± 12.74	78.95 ± 12.93	78.92 ± 12.81	**0.03**	NS
AI (%)	−14.63 ± 28.58	−22.33 ± 20.71	−12.99 ± 29.25	−20.83 ± 30.18	**0.01**	**0.01**
CAP (mm·Hg)	149.84 ± 32.73	130.18 ± 19.72	147.08 ± 30.55	128.99 ± 21.92	≤**0.001**	≤**0.001**

	DD ≤ 5		DD > 5		*p*3-value	*p*4-value
	Before LD	After LD	Before LD	After LD		
SBP (mm·Hg)	148.21 ± 21.13	136.40 ± 18.16	154.46 ± 37.83	139.71 ± 19.64	≤**0.001**	≤**0.001**
DBP (mm·Hg)	85.29 ± 12.79	77.24 ± 12.17	89.06 ± 22.46	77.86 ± 14.57	**0.02**	≤**0.001**
PP (mm·Hg)	62.91 ± 13.58	59.43 ± 14.13	65.57 ± 19.69	61.84 ± 11.68	≤**0.001**	**0.01**
HR (minute)	75.37 ± 12.78	77.72 ± 12.13	78.53 ± 12.59	79.68 ± 13.22	NS	NS
AI (%)	−14.12 ± 29.59	−20.22 ± 22.16	−13.59 ± 28.36	−22.73 ± 28.34	**0.02**	**0,01**
CAP (mm·Hg)	147.68 ± 29.14	129.41 ± 17.11	149.16 ± 33.67	129.75 ± 23.44	≤**0.001**	≤**0.001**

AI : augmentation index; CAP : central aortic pressure; DBP : diastolic blood pressure; DD : disease duration; HR : heart rate; H&Y : Hoehn and Yahr score; LD :  levodopa; NS: nonsignificant; PP: pulse pressure; SBP : systolic blood pressure; *p*_*1*_: H&*Y* < 3 before and after LD intake; *p*_*2*_: H&*Y* ≥ 3 before and after LD intake; *p*_*3*_: DD ≤ 5 before and after LD intake; *p*_*4*_: DD > 5 before and after LD intake; NS: nonsignificant. Bold numbers indicate significance.

**Table 4:** Parkinson's disease subtypes, nonmotor symptom, and their baseline hemodynamic and LD effects.

Disease Subtype	Tremor dominant type (n:38)		Akinetic rigid type (n:44)		*p-*value		
	Before LD	After LD	Before LD	After LD	*p1*	*p2*	*p3*
SBP (mm·Hg)	147.28 (±25.24)	141.89 (±15.04)	155.40 (±25.74)	150.12 (±159.65)	0.02	0.03	0.03
DBP (mm·Hg)	86.34 (±18.96)	77.45 (±13.87)	88.25 (±18.69)	81.23 (±16.79)	NS	0,02	0.02
PP (mm·Hg)	61.15 (±12.62)	55.12 (±10.65)	67.15 (±20.01)	62.56 (±18.12)	0.05	0.02	0.03
HR (minute)	77.05 (±14.46)	75.13 (±12.78)	77.15 (±11.12)	78.23 (±12.09)	NS	NS	NS
AI (%)	−7.43 (±30.72)	−9.45 (±31.67)	−19.35 (±26.01)	−20.55 (±267.34)	≤0.001	0,02	NS
CAP (mm·Hg)	148.37 (±32.47)	132.76 (±34.89)	148.60 (±31.06)	134.54 (±29.83)	NS	≤0.001	≤0.001

NMS	Incontinence (−) (n: 61)		Incontinence (+) (n: 21)		*p-*value		
SBP (mm·Hg)	146.83 (±24.71)	138.67 (±22.45)	165.61 (±43.41)	154.23 (±45.21)	0.01	0.02	0,03
DBP (mm·Hg)	85.95 (±17.27)	80.05 (±15.32)	91.47 (±22.40)	83.38 (±23.69)	NS	0.02	0,03
PP (mm·Hg)	61.01 (±12.96)	56.31 (±11.67)	74.14 (±23.55)	69.18 (±24–78)	≤0.001	0.03	0,02
HR (minute)	75.77 (±12.41)	76.02 (±13.45)	81.00 (±13.03)	80.23 (±14.69)	NS	NS	NS
AI (%)	−18.07 (±25.98)	−23.49 (±24.89)	−1.50 (±33.27)	−3.21 (±31.45)	0.02	≤0.001	≤0.001
CAP (mm·Hg)	145.11 (±31.16)	134.24 (±33.17)	158.33± (31.22)	135.67± (28.21)	0.01	≤0.001	≤0.001

NMS	Constipation (−) (n: 37)		Constipation (+) (n: 45)		*p-*value		
SBP (mm·Hg)	141.09 (±20.74)	134.41 (±21.34)	158.05 (±35.04)	142.12 (±31.35)	0.01	0,02	≤0.001
DBP (mm·Hg)	80.67 (±13.17)	74.47 (±11.26)	91.43 (±20.48)	86.23 (±18.29)	0.01	0,03	0,03
PP (mm·Hg)	60.67 (±13.30)	55.47 (±12.13)	66.62 (±18.90)	59.45 (±16.87)	NS	0,03	0,02
HR (minute)	75.03 (±10.76)	77.32 (±11.49)	78.37 (±13.69)	78.34 (±14.16)	NS	NS	NS
AI (%)	−19.71 (±27.13)	−24.36 (±29.21)	−10.25 (±29.36)	−15.12 (±28.86)	≤0.001	0,02	≤0.001
CAP (mm·Hg)	141.34 (±31.36)	129.37 (±33.45)	152.84 (±31.12)	134.67 (±29.82)	0.03	≤0.001	0,01

NMS	Orthostatic hypotension (−) (n: 51)		Orthostatic hypotension (+) (n: 31)		*p-*value		
SBP (mm·Hg)	151.94 (±30.87)	141.54 (±29.89)	151.16 (±32.74)	142.71 (±29.86)	NS	0,01	0,01
DBP (mm·Hg)	86.01 (±18.42)	81.45 (±17.32)	89.58 (±19.32)	82.36 (±17.45)	NS	0,04	0,02
PP (mm·Hg)	65.92 (±16.51)	59.38 (±15.76)	61.83 (±18.16)	55.28 (±16.15)	NS	0,03	0,01
HR (minute)	77.70 (±10.82)	78.32 (±11.49)	76.12 (±15.45)	77.56 (±16.78)	NS	NS	NS
AI (%)	−19.48 (±28.18)	−26.67 (±30.12)	−4.53 (±27.63)	−7.36 (±23.45)	0.02	0,02	≤0.001
CAP (mm·Hg)	147.35 (±30.57)	130.15 (±30.78)	150.38 (±33.46)	131.27 (±34.27)	NS	≤0.001	≤0.001

NMS	Psychosis (−) (n:70)		Psychosis (+) (n:12)		*p-*value		
SBP (mm·Hg)	154.67 (±31.77)	134.63 (±19.81)	131.11 (±11.55)	129.34 (±12.47)	≤0.001	0,01	NS
DBP (mm·Hg)	89.19 (±18.87)	79.81 (±14.34)	72.55 (±8.21)	73.23 (±9.15)	0.01	0,03	NS
PP (mm·Hg)	65.58 (±17.62)	57.61 (±12.45)	54.55 (±19.37)	53.97 (±18.36)	0.02	0,04	NS
HR (minute)	76.23 (±12.41)	77.39 (±11.25)	84.22 (±13.46)	85.21 (±14.19)	NS	NS	NS
AI (%)	−12.42 (±29.34)	−23.49 (±10.12)	−25.25 (±21.29)	−24.18 (±22.83)	0.01	0,01	NS
CAP (mm·Hg)	151.01 (±31.69)	132.41 (±31.78)	128.05 (±22.24)	121.79 (±22.34)	≤0.001	0,02	0,02

NMS	Dementia (−) (n:71)		Dementia (+) (n:11)		*p-*value		
SBP (mm·Hg)	148.02 (±24.25)	132.78 (±25.67)	155.00 (±56.34)	146.34 (±52.78)	0.02	≤0.001	0,02
DBP (mm·Hg)	86.28 (±16.45)	80.23 (±15.12)	94.36 (±29.70)	87.23 (±26.64)	0.04	0,03	0,01
PP (mm·Hg)	61.85 (±13.01)	56.32 (±11.56)	80.63 (±29.39)	78.24 (±28.45)	≤0.001	0,04	NS
HR (minute)	76.52 (±12.91)	77.64 (±13.91)	80.90 (±10.94)	85.98 (±11.63)	NS	NS	0,05
AI (%)	−14.66 (±27.68)	−18.71 (±26.32)	−11.46 (±35.94)	−12.71 (±34.67)	NS	0,03	NS
CAP (mm·Hg)	147.72 (±30.37)	136.89 (±28.53)	153.49 (±39.48)	141.45 (±36.81)	NS	0,02	0,02

AI : augmentation index; CAP : central aortic pressure; DBP: diastolic blood pressure; HR : heart rate; NMS : nonmotor symptom; NS: nonsignificant; PP :  pulse pressure; *p*1: TDP vs ARP before LD intake; NMS (−) vs NMS (+) before LD; *p*2: LD before vs LD after in TDP; LD before vs LD after NMS (−); *p*3: LD before vs LD after in ARP; LD before vs LD after of the NMS (+); SBP :  systolic blood pressure; (+): present; (−): absent. Bold numbers indicate significance.

## Data Availability

Data are available from the corresponding author upon reasonable request.

## References

[1] Laurent S., Kingwell B., Bank A., Weber M., Struijker-Boudier H. (2002). Clinical applications of arterial stiffness: therapeutics and pharmacology. *American Journal of Hypertension*.

[2] Brown L., Lorenz B., Erdmann E. (1985). The inotropic effects of dopamine and its precursor levodopa on isolated human ventricular myocardium. *Klinische Wochenschrift*.

[3] Noack C., Schroeder C., Heusser K., Lipp A. (2014). Cardiovascular effects of levodopa in Parkinson’s disease. *Parkinsonism & Related Disorders*.

[4] Oh Y. S., Kim J. S., Chung Y. A. (2013). Orthostatic hypotension, non-dipping and striatal dopamine in Parkinson disease. *Neurological Sciences*.

[5] Kim J. S., Oh Y. S., Lee K. S. (2014). Carotid artery thickening and neurocirculatory abnormalities in de novo Parkinson disease. *Journal of Neural Transmission*.

[6] Goldstein D. S., Holmes C., Cannon R. O., Eisenhofer G., Kopin I. J. (1997). Sympathetic cardioneuropathy in dysautonomias. *New England Journal of Medicine*.

[7] Jain S., Goldstein D. S. (2012). Cardiovascular dysautonomia in Parkinson disease: from pathophysiology to pathogenesis. *Neurobiology of Disease*.

[8] Lee H. Y., Oh B. H. (2010). Aging and arterial stiffness. *Circulation Journal*.

[9] Rosenwasser R. F., Shah N. K., Smith S. M. (2014). Baseline predictors of central aortic blood pressure: a PEAR substudy. *Journal of the American Society of Hypertension*.

[10] Gaszner B., Lenkey Z., İllyes M. (2012). Comparison of aortic and carotid arterial stiffness parameters in patients with verified coronary artery disease. *Clinical Cardiology*.

[11] Mulders T. A., van den Bogaard B., Bakker A. (2012). Arterial stiffness is increased in families with premature coronary artery disease. *Heart*.

[12] Fantin F., Mattocks A., Bulpitt C. J., Banya W., Rajkumar C. (2006). Is augmentation index a good measure of vascular stiffness in the elderly?. *Age and Ageing*.

[13] Yamashina A., Tomiyama H., Takeda K. (2002). Validity, reproducibility, and clinical significance of noninvasive brachial-ankle pulse wave velocity measurement. *Hypertension Research*.

[14] Tomiyama H., Yamashina A. (2010). Non-invasive vascular function tests: their pathophysiological background and clinical application. *Circulation Journal*.

[15] Williams B., Lacy P. S., Thom S. M. (2006). The CAFE investigators, CAFE steering committee and writing committee, CAFE investigators, anglo-scandinavian cardiac outcomes trial investigators, CAFE steering committee and writing committee, ‘‘Differential impact of blood pressure-lowering drugs on central aortic pressure and clinical outcomes: principal results of the conduit artery function evaluation (CAFE) study. *Circulation*.

[16] De Simone G., Roman M. J., Alderman M. H., Galderisi M., de Divitiis O., Devereux R. B. (2005). Is high pulse pressure a marker of preclinical cardiovascular disease?. *Hypertension*.

[17] Park J. H., Han S. W., Baik J. S. (2017). A comparative study of central hemodynamics in Parkinson’s disease. *Journal of Movement Disorders*.

[18] Amino T., Orimo S., Itoh Y., Takahashi A., Uchihara T., Mizusawa H. (2006). Profound cardiac sympathetic denervation occurs in Parkinson disease. *Brain Pathology*.

[19] Goldstein D. S. (2003). ‘Dysautonomia in Parkinson’s disease: neurocardiological abnormalities. *The Lancet Neurology*.

[20] Solla P., Cadeddu C., Cannas A. (2015). Heart rate variability shows different cardiovascular modulation in Parkinson’s disease patients with tremor dominant subtype compared to those with akinetic rigid dominant subtype. *Journal of Neural Transmission*.

[21] Spiegel J., Hellwig D., Farmakis G. (2007). Myocardial sympathetic degeneration correlates with clinical phenotype of Parkinson’s disease. *Movement Disorders*.

[22] Tulba D., Cozma L., Balanescu P., Buzea A., Băicuș C., Popescu B. O. (2021). Blood pressure patterns in patients with Parkinson’s disease: a systematic review. *Journal of Personalized Medicine*.

[23] Goldstein D. S., Holmes C., Bentho O. (2008). Biomarkers to detect central dopamine deficiency and distinguish Parkinson disease from multiple system atrophy. *Parkinsonism & Related Disorders*.

[24] Chiaravalloti A., Stefani A., Di Biagio D. (2013). Cardiac sympathetic denervation is not related to nigrostriatal degeneration in Parkinson’s disease. *Annals of Nuclear Medicine*.

[25] Kim J. S., Lee S. H., Oh Y. S. (2016). Cardiovascular autonomic dysfunction in mild and advanced Parkinson’s disease. *Journal of Movement Disorders*.

[26] Pierzchlinska A., Kwaśniak-Butowska M., Sławek J., Drozdzik M., Białecka M. (2021). Arterial blood pressure variability and other vascular factors contribution to the cognitive decline in Parkinson’s disease. *Molecules*.

[27] Goldstein D. S., SEwell L., Sharabi Y. (2011). Autonomic dysfunction in PD: a window to early detection?. *Journal of the Neurological Sciences*.

[28] Orimo S., Oka T., Mura H. (2002). Sympathetic cardiac denervation in Parkinson’s disease and pure autonomic failure but not in multiple system atrophy. *Journal of Neurology, Neurosurgery & Psychiatry*.

[29] Goldstein D. S., Holmes C., Sewell L., Park M. Y., Sharabi Y. (2012). Sympathetic noradrenergic before striatal dopaminergic denervation: relevance to Braak staging of synucleinopathy. *Clinical Autonomic Research*.

[30] Kim J. S., Lee S. H., Oh Y. S. (2017). Arterial stiffness and cardiovascular autonomic dysfunction in patients with Parkinson’s disease. *Neurodegenerative Diseases*.

[31] Chiaravalloti A., Stefani A., Tavolozza M. (2012). Different patterns of cardiac sympathetic denervation in tremor-type compared to akinetic-rigid-type Parkinson’s disease: molecular imaging with ^123^I-MIBG. *Molecular Medicine Reports*.

